# Structural, Electronic, and Mechanical Properties of Zr_2_SeB and Zr_2_SeN from First-Principle Investigations

**DOI:** 10.3390/ma16155455

**Published:** 2023-08-03

**Authors:** Xiaojing Bai, Ke Chen, Kan Luo, Nianxiang Qiu, Qing Huang, Qi Han, Haijing Liang, Xiaohong Zhang, Chengying Bai

**Affiliations:** 1 School of Materials Science and Engineering, Anyang Institute of Technology, Anyang 455000, China; 2 Engineering Laboratory of Advanced Energy Materials, Ningbo Institute of Materials Technology and Engineering, Chinese Academy of Sciences, Ningbo 315201, China; 3 College of Science & Technology, Ningbo University, Ningbo 315300, China; 4 School of Material Science and Engineering, China University of Petroleum, Qingdao 266580, China; 5 Key Laboratory of Superlight Materials and Surface Technology, Ministry of Education, College of Materials Science and Chemical Engineering, Harbin Engineering University, Harbin 150001, China

**Keywords:** first principles, Zr_2_SeB/N, electronic properties, mechanical properties

## Abstract

MAX phases have exhibited diverse physical properties, inspiring their promising applications in several important research fields. The introduction of a chalcogen atom into a phase of MAX has further facilitated the modulation of their physical properties and the extension of MAX family diversity. The physical characteristics of the novel chalcogen-containing MAX 211 phase Zr_2_SeB and Zr_2_SeN have been systematically investigated. The present investigation is conducted from a multi-faceted perspective that encompasses the stability, electronic structure, and mechanical properties of the system, via the employment of the first-principles density functional theory methodology. By replacing C with B/N in the chalcogen-containing MAX phase, it has been shown that their corresponding mechanical properties are appropriately tuned, which may offer a way to design novel MAX phase materials with enriched properties. In order to assess the dynamical and mechanical stability of the systems under investigation, a thorough evaluation has been carried out based on the analysis of phonon dispersions and elastic constants conditions. The predicted results reveal a strong interaction between zirconium and boron or nitrogen within the structures of Zr_2_SeB and Zr_2_SeN. The calculated band structures and electronic density of states for Zr_2_SeB and Zr_2_SeN demonstrate their metallic nature and anisotropic conductivity. The theoretically estimated Pugh and Poisson ratios imply that these phases are characterized by brittleness.

## 1. Introduction

The family of materials denoted as the MAX phases is a subject of great interest within the scientific community, with a general formulation expressed as M*_n_*_+1_AX*_n_*, where *n* can take on values of 1, 2, or 3. In this expression, M denotes an early transition metal, A typically represents an A-group element, and X is predominantly characterized by C or N. The origins of this family of materials can be traced back to the pioneering research of Nowotny et al. in the 1960s [1,2,3,4]. Until the 1990s, the interest in MAX phases was reignited by Barsoum et al. [5,6], who revealed their exceptional properties. These materials exhibit metallic like characteristics such as high electrical and thermal conductivity, as well as machinability and mechanical strength. Additionally, they possess exceptional mechanical properties at high temperatures and exhibit highly sensible corrosion and reaction resistance, which are similar to those of ceramics [7]. All these unique properties can be attributed to their nano-layered structures, where elemental A in the style of a single atomic layer is situated between M_n+1_X_n_ sheets. Furthermore, the bond of M-A plays a pivotal role in determining the chemical and physical characteristics. This distinctive set of properties has resulted in the identification of over 150 MAX phases that can be utilized in various applications [8,9].

Given the growing demand for MAX phase materials, researchers have been exploring ways to enhance their structural versatility and performance flexibility. This has led to the development of novel MAX phase compounds or the recombination of M, A, and/or X elements in existing structures [8]. Recently, boron (an element with an atomic number of five) was introduced as an additional X element, resulting in an expanded range of MAX phases. The remarkable physical and chemical properties of boron and corresponding compounds make them highly desirable for high-temperature applications, thereby creating a pressing need for boride MAX phases [10]. As such, the boride MAX phase, much like its conventional MAX phase counterparts, has garnered significant research attention [11,12,13,14,15,16,17]. Khazaei [11] systematically investigated the structure and properties of the Sc_2_AlB, Ti_2_AlB, Cr_2_AlB, Zr_2_AlB, and Nb_2_AlB MAX phase borides. The MAX phase borides Ti_2_AlB, Ti_2_GaB, and Ti_2_InB have also been investigated via theoretical approaches [13]. In addition, the chalcogen-containing MAX phases with more robust mechanical properties received greater attention than the corresponding aluminum-containing MAX phases [18]. Currently, the MAX phases with sulfur elements at A sites are limited to Ti_2_SC, Zr_2_SC, Hf_2_SC, Nb_2_SC, and M_2_SB (M = Zr, Hf, Nb) [19]. The experimental realization of Se occupying the A site in a Zr_2_SeC MAX phase has expanded the family of nano-laminated ternary carbides [20]. Recently, the DFT method was utilized to investigate the physical characteristics of novel chalcogen-containing MAX phases, Hf_2_SeC and Zr_2_SeC, for high-temperature applications [21,22]. This study aims to have an in-depth study of the effect of replacing carbon with boron or nitrogen as the X element on the crystal lattice constant, electronic structure, and several physical properties of Se-containing MAX phases, which would enrich the materials’ properties and extend their potential applications. In addition, to obtain complete image of chalcogen-containing MAX phases, the electronic properties of Zr_2_SeC was calculated for comprehensive comparison. In this work, the predicted results of the B/N atom replacing the C atom in the ternary 211 MAX-phase nano-laminates essentially hold the potential to enhance the properties of the MAX phase materials and broaden their applications in various fields.

## 2. Computational Details

Throughout the whole work, density functional theory (DFT) [23] calculations were performed using the Cambridge Serial Total Energy Package (CASTEP) [24]. The electronic exchange–correlation interaction was described using the generalized gradient approximation [25] with the Perdew–Burke–Ernzerh [26] (GGA-PBE) functional. The elemental core and valance electrons were implemented by norm-conserving pseudopotentials and a plane-wave basis functional with kinetic energy cutoff of 520 eV. The main calculated electronic configurations were Zr: 4s^2^4p^6^4d^2^5s^2^, Se: 4s^2^4p^4^, N: 2s^2^2p^3^, and B: 2s^2^2p^1^. To optimize the geometric structure and cell structure, the Broyden–Fletcher–Goldfarb–Shanno (BFGS) minimization scheme was chosen to equilibrize the structure. Throughout the entire self-consistent field (SCF) calculation process, the difference of total energy [27], Hellman–Feynman forces on each atom, atomic displacements, and the stresses were less than 1.0 × 10^−7^ eV/atom, 0.002 eV/Å, 1 × 10^−3^ Å, and 0.05 GPa, respectively, to achieve the convergence threshold.

## 3. Results and Discussions

### 3.1. Analysis of Crystal Structure of Zr_2_SeX (X = B, N)

**Figure 1:**
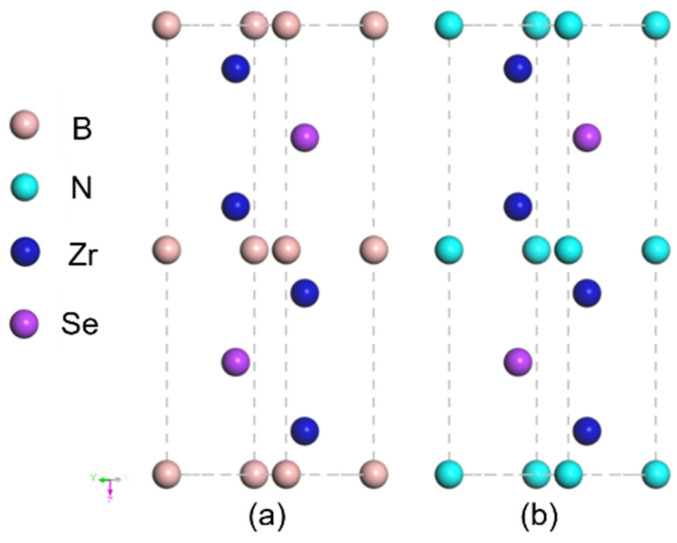
The crystal structures of Zr_2_SeB (**a**) and Zr_2_SeN (**b**).

The atomic models of Zr_2_SeB and Zr_2_SeN cells were first constructed for the optimization of geometric configuration before the investigation of the electronic structure and their respective physical properties. As depicted in Figure 1, Zr_2_SeX (with B or N located at the X site) with a hexagonal crystal structure and space group of P6_3_/mmc, has eight atoms (four Zr atoms, two X atoms, and two Se atoms) in a unit cell, which is identical to that of the reported Zr_2_SeC.

Based on the input parameters utilized in this investigation, the optimized lattice constants of the Zr_2_SeC cells exhibit strong agreement with similar structures (Table 1), alongside corresponding experimental and theoretical values. Moreover, we can see that our results of Zr_2_SeB and Zr_2_SeN are in good agreement with other reported data [28]. After the identification and comparison of calculation results, the lattice consists of the a and c values of Zr 211 MAX compounds, and the Se, at the A site, is larger than the S, which can be ascribed to the atomic size. Specifically, the calculated values of a and c for Zr_2_SeC show a 0.63% and 0.72% increase, respectively, relative to prior theoretical results.

**Figure 2:**
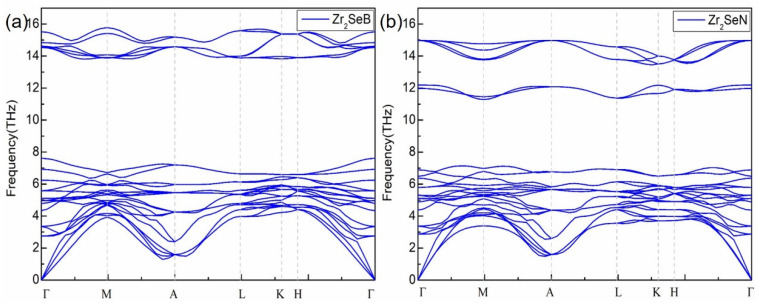
The phono spectrum of Zr_2_SeB (**a**) and Zr_2_SeN (**b**).

The dynamic stabilities of both configurations of Zr_2_SeB and Zr_2_SeN were revealed by the theoretical calculations of the phonon dispersions [29], as presented in Figure 2. A unit cell of Zr_2_SeB or Zr_2_SeN has eight atoms and thus has twenty-four phonon branches (three acoustic and twenty-one optical branches). They are labeled according to their symmetries at the Γ point: TA and LA modes are the in-plane transverse and longitudinal acoustic modes, the vibration planes of these two phonons are along the ab plane direction. The vibration plane of the other transverse acoustic branch (ZA) phonon is perpendicular to the ab plane. The slopes indicate their group velocities, and the slope of LA is the largest, while the slope of ZA is smaller than that of LA and TA. In addition, the irreducible representation can be classified as Γoptical = 2E1g + 4E2g + A1g + 4E1u + 2A2u + 4E2u + 2B2g + 2B1u. The E1g, E2g, and A1g modes are the Raman active vibration modes, and the E1u and A2u modes are the IR active vibration modes. Notably, the phonon frequencies of the Zr_2_SeB structure were observed to be considerably higher than those of the corresponding Zr_2_SeN structure, which could be ascribed to a lighter mass of the boron element than that of nitrogen atom.

### 3.2. Electronic Properties

**Figure 3:**
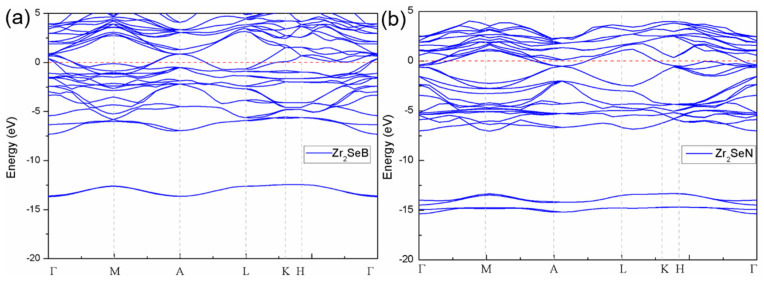
Electronic band structures of Zr_2_SeB (**a**) and Zr_2_SeN (**b**).

On the basis of symmetry of hexagonal crystal system, to optimize the calculation process of electronic properties of two models, a high symmetry path of G-A-L-K-H towards the Brillouin zone was adopted to investigate the electronic band structures of Zr_2_SeB and Zr_2_SeN (Figure 3). Due to their comparable structures, there is a certain degree of similarity in their band structures. The electronic energy bands of both configurations, as shown in Figure 3, overlap near the Fermi level (EF), indicating a metal-like conductivity that is similar to Zr_2_SeC.

According to the band structure (Figure 3), it can be seen that electronic conduction is naturally anisotropic. In the K-L and H-K directions, the energy dispersion with a unit area is small along the c-direction. Conversely, conductivity within the basal plane is demonstrated by G-M, and the L-A direction is higher than that within the basal plane, indicated by K-L and H-K directions with a unit area in the c-direction. Thus, it follows that Zr_2_SeB and Zr_2_SeN exhibit higher basal plane conductivity than the c-directional conductivity, a characteristic analogous to most traditional MAX phases reported in the literature [18].

**Figure 4:**
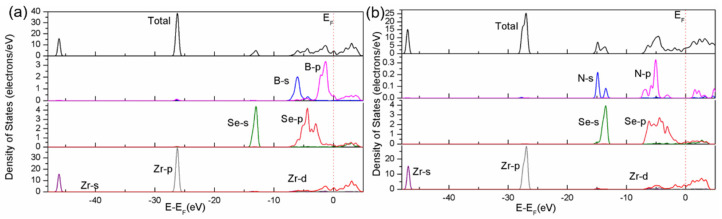
Calculated Density of States of Zr_2_SeB (**a**) and Zr_2_SeN (**b**).

The density of states (DOS) for Zr_2_SeB and Zr_2_SeN is depicted in Figure 4. The high degree of similarity between the electronic bands of the two materials demonstrates that the DOS diagrams of then would be similar consequently. In agreement with the analysis results of energy bands, Zr_2_SeB and Zr_2_SeN belong to electronic conductors, according to the several orbit states (such as B-p and Zr-d) that occupy the Fermi level. The contribution from the different states of Zr, Se, N, and B to the total DOS is confirmed from the partial DOS (PDOS) diagrams. For instance, Zr’s 4d orbit state provides the dominant contribution around the Fermi level, corresponding to the electronic structure of the reported Zr_2_SeC [20,21]. Neither N nor Se contributes to the DOS at the Fermi level, which is consistent with prior experimental and theoretical results [18,21]. Moreover, obvious hybridization could be observed from the PDOS diagrams in both Zr_2_SeB and Zr_2_SeN. In Figure 4a, as for Zr_2_SeB, B’s 2p state splits, owing to strong hybridization with Zr’s 4d state. In Zr_2_SeN, similarly, a strong hybridization between N’s 2p state and Zr’s 4d state is observed in the energy range from −7.5 to −4.5 eV. Furthermore, in both Zr_2_SeN and Zr_2_SeB, it is non-negligible that Se’s p state is hybridized with Zr’s d states. 

### 3.3. Mechanical Properties

Dynamic characteristics are significant for evaluating a material’s performance. The elastic constants predict these traits and behaviors, which can represent important macroscopic properties. Herein, the stress–strain method was applied to calculate the various elastic constants of Zr_2_SeB and Zr_2_SeN, and corresponding values are presented in Table 2. As the MAX phases are hexagonal in crystal symmetry, they possess six elastic constants $C_{ij}\left(C_{11},C_{12},C_{13},C_{33},C_{44}-C_{55},C_{66}\right)$ Of these, $C_{66}$ is dependent $\left(C_{66}=\left(C_{11}-C_{12}\right)/2\right)$. The mechanical stability of a material under load is a critical factor in practical applications, and the stability conditions [30] for hexagonal systems dictate that $C_{11}>\left|C_{12}\right|$, $\left(C_{11}+C_{12}\right)C_{33}>2C_{13}^{2}$, $C_{44}>0$, and $C_{66}>0$. Consequently, the mechanical stability of Zr_2_SeB and Zr_2_SeN should be fulfilled by the four aforementioned conditions. From the obtained elastic constants, other important parameters can also be calculated, such as the bulk modulus, B; the shear modulus, G; Young’s modulus, E; Poisson’s ratio, σ; and Debye’s temperature, $\theta_{D}$, using relevant equations with the software [31,32,33].

(1) $$B_{R}=\frac{C_{33}\left(C_{11}+C_{12}\right)-2C_{13}^{2}}{C_{11}+C_{12}+2C_{33}-4C_{13}}$$

(2) $$B_{V}=\frac{1}{9}\left[2\left(C_{11}+C_{12}\right)+C_{33}+4C_{13}\right]$$

(3) $$G_{R}=\frac{5}{2}\frac{C_{44}\left(C_{11}-C_{12}\right)\left[C_{33}\left(C_{11}+C_{12}\right)-2C_{13}^{2}\right]}{3B_{V}C_{44}\left(C_{11}-C_{12}\right)+\left\{2C_{44}+\left(C_{11}-C_{12}\right)\left[C_{33}\left(C_{11}+C_{12}\right)-2C_{13}^{2}\right]\right\}}$$

(4) $$G_{V}=\frac{1}{30}\left[C_{11}+C_{12}+2C_{33}-4C_{13}+12C_{44}+6\left(C_{11}-C_{12}\right)\right]$$

(5) $$B=\frac{1}{2}(B_{R}+B_{V})$$

(6) $$G=\frac{1}{2}(G_{R}+G_{V})$$

(7) $$E=\frac{9BG}{3B+G}$$

(8) $$\sigma =\frac{3B-2G}{2\left(3B+G\right)}$$

(9) $$v_{l}=\sqrt{\frac{3B+4G}{3\rho }}$$

(10) $$v_{t}=\sqrt{\frac{G}{\rho }}$$

(11) $$v_{m}={\left[\frac{1}{3}\left(\frac{2}{v_{t}^{3}}+\frac{1}{v_{l}^{3}}\right)\right]}^{-1/3}$$

(12) $$\theta_{D}=\frac{h}{k}{\left[\frac{3n}{4\pi }\left(\frac{N_{A}\rho }{M}\right)\right]}^{1/3}v_{m}$$

Here, $v_{l}$ and $v_{t}$ represent longitudinal and transverse sound velocities, respectively. ρ is the density of the cell, $v_{m}$ is the averaged sound velocity, h is Planck’s constant, and k is Boltzmann’s constant.

Elastic constants provide crucial insights into bonding behaviors across different crystallographic planes. Specifically, the Zr_2_SeX (*X* = C, B, N) compounds exhibit greater compression along the *c*-axis than the *a*-axis. This observation is supported by the lattice parameters of the MAX phases, which indicates a preferential compression along the c-axis, as opposed to that along the a-axis. This trend is a common feature of MAX phases and is reflected in their elastic anisotropic characteristics.

To analyze and estimate the brittleness or toughness of a material, Poisson’s ratio (v), as a critical parameter, is usually tested. Traditionally, the transition value of 0.26 is the threshold for evaluating whether a material is brittle or ductile. As shown in Table 2, the Zr_2_SeN and Zr_2_SeB MAX phases are relatively brittle when compared to Zr_2_SeC and Zr_2_SB. Furthermore, Pugh’s ratio is a valuable tool in predicting ductile or brittle failure modes by examining the ratio of bulk to shear moduli. A critical value of 1.75 is used to classify materials as either ductile or brittle, with a B/G value greater than 1.75, indicating a ductile character. The MAX phases of Zr_2_SeN and Zr_2_SeB are classified as brittle, similar to the previously reported Zr_2_SeC.

It can be inferred that Zr_2_SeB possesses higher B and G values when compared to Zr_2_SeN (Table 2). This observation indicates that Zr_2_SeB necessitates greater pressure than Zr_2_SeN for bulk and plastic deformation. Furthermore, the E values of Zr_2_SeB surpass those of Zr_2_SeN, suggesting that Zr_2_SeB exhibits greater hardness than Zr_2_SeN. Moreover, $C_{44}$, an important indicator of material hardness, exhibits a strong correlation with hardness in comparison to other elasticity moduli. Consequently, Zr_2_SeB is expected to possess a higher $C_{44}$ than Zr_2_SeN, thereby enhancing its hardness. In contrast, Zr_2_SeN’s hardness is lower than Zr_2_SeC and Zr_2_SeB. These findings offer new clues for tuning the X-composition of the substituted MAX phase materials, potentially leading to improved performance in various applications.

Debye’s temperature, $\theta_{D}$, helps to predict the application of the material at high temperatures. Using the Anderson model, the $\theta_{D}$ of Zr_2_SeN and Zr_2_SeB are 499 K and 498 K, respectively, which are lower than that of Zr_2_SeC (512 K, calculated using Anderson’s model; 679 K, calculated via the quasi-harmonic Debye model [21]). The $\theta_{D}$ of Zr_2_SeB (498 K), calculated via Anderson’s model, is lower than that of Zr_2_SB (540 K, obtained using the quasi-harmonic Debye model [18]). Comparable results are seen in other carbides MAX 211 phases, such as the reported Zr_2_SC, Hf_2_SB/C, and Nb_2_SC/B [18,34]. In the high-temperature applications of Zr_2_SeN and Zr_2_SeB, such as thermal barrier coating (TBC), the Debye temperatures are required.

**Figure 5:**
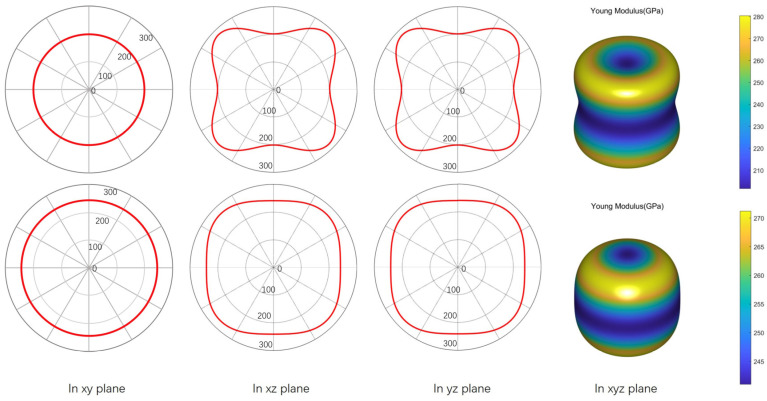
Directional variation in Young’s modulus (E) of Zr_2_SeB (the first line) and Zr_2_SeN (the second line).

**Figure 6:**
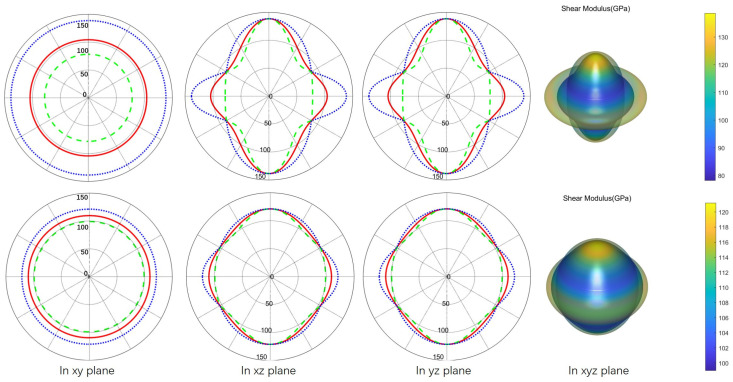
Directional variation in shear modulus (G) of Zr_2_SeB (the first line) and Zr_2_SeN (the second line).

Mechanical anisotropy is one of the non-negligible factors that are closely related to the potential applications of functional materials. For example, in practical applications, the material itself may produce micro-cracks or undergo deformation in different directions, which is limited by their intrinsic mechanical properties. Thus, the mechanical anisotropies of Zr_2_SeB and Zr_2_SeN are investigated, and corresponding data were recorded in the forms of 2D and 3D. Visually, as shown in Figure 5 and Figure 6, Young’s modulus and shear modulus of Zr_2_SeB and Zr_2_SeN are direction-dependent. Usually, in terms of measurement of elastic moduli, the spherical shape of the curved surface in 3D and the circular shape of plots in 2D indicate the isotropic mechanical behavior of solids. However, deviations from spherical/circular symmetry or symmetry breaking indicate that the mechanical properties of the measured object are anisotropic. Meanwhile, the degree of anisotropy of the elastic moduli of a substance is measured through the amount of deviation from a perfect sphere/circle. Figure 5 shows the directional dependence of E for Zr_2_SeB and Zr_2_SeN. As can be seen in Figure 5, E is isotropic in the xy plane, and its plot shape is uniformly circular. In contrast, E is anisotropic in the xz and yz planes. It can be seen in Figure 6 that the G of Zr_2_SeB and Zr_2_SeN does not change direction on the xy plane, and the two-dimensional graph is uniformly circular but changes direction on both the xz and yz planes. These are essentially identical to the symmetry of hexagonal crystals and are consistent with the findings of M.A. Hadi et al [18].

By mechanical analysis, the Zr_2_SeN and Zr_2_SeB phases can be tentatively identified as elastically anisotropic. The three shear anisotropy coefficients that depend on the hexagonal crystal of $C_{ij}$ [35], which quantify the degree of elastic anisotropy, can be obtained as follows [36]:

(13) $$A_{1}=\frac{\left(C_{11}+C_{12}+2C_{33}-4C_{13}\right)}{6C_{44}}$$

which is associated with the {100} shear planes in the 〈011〉 and 〈010〉 directions;

(14) $$A_{2}=\frac{2C_{44}}{C_{11}-C_{12}}$$

which is related to the {010} shear planes in the 〈101〉 and 〈001〉 directions;

(15) $$A_{3}=\frac{\left(C_{11}+C_{12}+2C_{33}-4C_{13}\right)}{3\left(C_{11}-C_{12}\right)}$$

which denotes the shear anisotropy that occurs in the 001 shear planes in the 〈110〉 and 〈010〉 directions. As is well known, in anisotropic crystals, $A_{i}\left(i=1,2,3\right)$ would have a value other than unity. In contrast, all factors of $A_{i}\left(i=1,2,3\right)$ would have a unit value in all isotropic systems [37]. Moreover, the deviation of $A_{i}$ from unity ($\Delta A_{i}$) could determine the degree of elastic anisotropy in the shear. Subsequently, Zr_2_SeN and Zr_2_SeB exhibit elastically anisotropic in the shear (Table 3). Moreover, regarding the specific evaluation of elastic anisotropy for the hexagonal crystal, there is another anisotropy factor that is obtained from $C_{ij}$, $k_{c}/k_{a}=\left(C_{11}+C_{12}-2C_{13}\right)/\left(C_{33}-C_{13}\right)$ [38]. In the formula, $k_{a}$ and $k_{c}$, respectively, represent the linear compressibility coefficients along the *a* and *c* axes. All the values of $k_{c}/k_{a}$ (Table 3), which are different from unity ($\Delta k_{c}/k_{a}$), demonstrating the degree of anisotropy of Zr_2_SeN and Zr_2_SeB under a linear compression in the a and c directions.

Hill’s theory proposed a proportional relationship between difference between $B_{V}$ and $B_{R}$, and the elastic anisotropy level of crystals. The same relationship is appropriate for the difference between $G_{V}$ and $G_{R}$ as well. Then, the percentage of anisotropy factors $A_{B}$ and $A_{G}$ can be calculated as follows:

(16) $$A_{B}=\frac{B_{V}-B_{R}}{B_{V}+B_{R}}\times 100\%$$

(17) $$A_{G}=\frac{G_{V}-G_{R}}{G_{V}+G_{R}}\times 100\%$$

Considering the compressibility and shear, these two coefficients are assigned zero values for fully isotropic crystals. We can conclude that the Zr_2_SeB and Zr_2_SeN phases are anisotropic, as confirmed by the results in Table 3. The current knowledge about the anisotropy of Zr_2_SeN and Zr_2_SeB contributes to their mechanical stability during specific physical processes, including the occurrence of plastic deformation and the generation of microscale cracks.

## 4. Conclusions

In summary, the electronic structure and several mechanical properties of two chalcogen-containing ternary MAX phases, Zr_2_SeB and Zr_2_SeN, were investigated systematically via DFT calculations. The lattice parameters of Zr_2_SeB and Zr_2_SeN are consistent with those of Zr_2_SeC and Zr_2_SB, and these MAX phases exhibit dynamical and mechanical stability. Through the analyses of band structure and the density of states, the electronic character of Zr_2_SeB and Zr_2_SeN is identified as a metal, which is consistent with that of the conventional MAX phases. Furthermore, we investigate the mechanical properties of Zr_2_SeB and Zr_2_SeN and compare them with those of Zr_2_SB and Zr_2_SeC, obtained from previous studies. The intrinsic anisotropy of Zr_2_SeB and Zr_2_SeN in bonding strength along the a- and c-axis is revealed via calculations, and the mechanical properties of Zr_2_SeB and Zr_2_SeN, such as elastic anisotropic characteristics, brittleness, hardness, and mechanical stability, are comparable with those of other prior-reported chalcogenide–MAX phases. From the inspiration of this work, it is worth noting that the mechanical properties and electronic structure of MAX phases can be obviously modulated by designing the X element rationally. Thereby, the construction of two MAX-phase materials in this work and corresponding calculations provide an effective strategy for selecting and optimizing MAX phases towards broader applications.

## Figures and Tables

**Table 1:** The lattice parameter data (*a*, *c*) for Zr_2_SeX (X = C, B, N).

	Methods	*a* (Å)	*c* (Å)	*c*/*a*	
Zr_2_SeC	GGA-PBE	3.487	12.631	3.622	This work
Zr_2_SeB	GGA-PBE	3.578	12.734	3.559	This work
Zr_2_SeN	GGA-PBE	3.478	12.371	3.557	This work
Zr_2_SeC	Experiment	3.491	12.556	3.615	[20]
Zr_2_SeC	GGA-PBE	3.465	12.540	3.618	[21]
Zr_2_SB	GGA-PBE	3.516	12.313	3.502	[18]
Zr_2_SC	GGA-PBE	3.420	12.205	3.568	[18]

**Table 2:** The calculated mechanical properties ($C_{ij}$, B, G, and E in GPa, $\theta_{D}$ in K, σ) of Zr_2_SeB and Zr_2_SeN compared with other theoretical and experimental results.

MAX	$C_{11}$	$C_{12}$	$C_{13}$	$C_{33}$	$C_{44}$	$C_{66}$	B	G	E	σ	$\theta_{D}$	B/G	Ref.
Zr_2_SeC	276	75	93	289	126	100	151	109	264	0.20	512	1.38	[20]
Zr_2_SeB	264	107	105	303	138	78	162	106	260	0.23	498	1.54	This work
Zr_2_SeN	274	75	100	279	121	99	153	105	256	0.22	499	1.45	This work

**Table 3:** Elastic anisotropy factors for Zr_2_SeX (*X* = C, B, N) MAX phases.

MAX	$A_{1}$	$A_{2}$	$A_{3}$	$k_{c}/k_{a}$	$A_{B}$	$A_{G}$	Ref.
Zr_2_SeC	0.73	1.57	1.14	0.84	0.64	0.30	[21]
Zr_2_SB	0.85	1.35	1.14	0.91	0.04	0.90	[18]
Zr_2_SeB	0.67	1.76	1.18	0.81	0.63	0.30	This work
Zr_2_SeN	0.70	1.22	0.85	0.83	0.63	0.31	This work

## Data Availability

Data will be made available on request.
